# Analysis of whole genome-wide microRNA transcriptome profiling in invasive pituitary adenomas and non-invasive pituitary adenomas

**DOI:** 10.1186/s41016-019-0177-4

**Published:** 2019-12-02

**Authors:** Chao Zhang, Yuan Qian, Yisheng Qiao, Yao Li, Wei Wang, Junjun Li, Xingli Deng

**Affiliations:** 1 Department of Neurological Surgery, 1st Affiliated Hospital of Kunming Medical University, Kunming, China; 2 Yunnan Key Laboratory of Laboratory Medicine, Yunnan Engineering Technology Center of Digestive disease, 1st Affiliated Hospital of Kunming Medical University, Kunming, China; 3 Genetic Diagnosis Center, Kunming City Maternal and Child Health Hospital, Kunming, China

**Keywords:** Pituitary adenoma, Invasive, MicroRNA, Transcriptome

## Abstract

**Background:**

Dysregulation of microRNAs (miRNAs) plays a critical role during the occurrence and progress of pituitary adenomas (PAs). However, the roles of miRNAs in the invasiveness of PA are poorly understood. This study aims to more comprehensively and specific define the relationship between altered miRNA and PA invasion.

**Methods:**

The differential expression of miRNAs (DEMs) between invasive PAs (IPAs) and non-invasive PAs (NPAs) was explored by RNA sequencing and which functions were analyzed by gene ontology (GO) as well as Kyoto Encyclopedia of Genes and Genomes (KEGG). The miRNA-mRNA network was predicted with bioinformatics.

**Results:**

We identified 31 upregulated miRNAs and 24 downregulated miRNAs in IPAs compared with NPAs. GO analysis and KEGG pathway analysis showed the DEMs were mainly associated with cell proliferation and cell cycle pathway. In addition, on the count of predicted miRNA-mRNA network, two hub miRNAs were identified.

**Conclusions:**

Our results demonstrate the miRNA-mRNA network in detail, which suggest that miRNA may be a promising target in diagnosis and therapy for IPAs.

## Background

PA is one of the most common intracranial tumors with an incidence of 10–15% [1]. Generally, PAs are considered benign, but some of them are invasive, which invade the adjacent structures such as sphenoid sinus, cavernous sinus, and diaphragma sellae [2–4]. Therefore, IPAs are not only more difficult to complete surgical resection, but also more likely to recurrence after surgery.

MiRNAs are single-strand non-coding RNAs of approximately 19–23 nt, which regulate gene expression at the post-transcriptional level [5, 6], and they can also act as tumor suppressor genes or oncogenes in various tumors [7]. For instance, miR-193b exerts tumor suppressive effects in human acute myeloid leukemia by inducing tumor cell apoptosis and G1/S arrest [8], while miR-210-3p plays an oncogene role in prostate cancer by promoting cancer cell epithelial–mesenchymal transition and bone metastasis via NF-κB signaling pathway [9]. Altered expression of many miRNAs has been described in PAs, and specific miRNA signatures are related to clinical and therapeutic characteristics of the tumors [10]. However, comprehensive and specific researches of relationships between miRNAs and invasiveness of PAs are still rare.

In order to better understand the mechanism of invasiveness in PAs, it is necessary to clarify the miRNA regulatory network in IPAs. In this study, we detected DEMs in IPAs and NPAs by RNA sequencing, and established the co-expression network contain miRNAs and predicted target genes by Cytoscape. In addition, the expression of the most upregulated miR-665 and the most downregulated miR-149-3p in IPAs was screen out. Moreover, we explained the potential functions of the two key miRNAs in invasive behavior of PAs by GO analysis and KEGG pathway analysis.

### Methods

### Patients and samples

Seven tumor samples were obtained from patients with PAs who underwent operation at the Department of Neurosurgery, 1st Affiliated Hospital of Kunming Medical University for identification of miRNAs by high-throughput sequencing. None of these patients has been received radiotherapy or chemotherapy before surgery. Tumor samples were divided into 2 groups according to invasive behavior proved by surgical findings and pathology: IPA and NPA. All patients were informed according to inform consent approved by the Ethics Boardof the 1st Affiliated Hospital of Kunming Medical University. Immediately following separation, the fresh tumor samples were placed in sterile, RNase-free 2.0-mL cryotubes. Then, samples were soaked in Trizol and stored at − 80 °C for following analysis.

### RNA isolation, library preparation, and sequencing analysis

Total RNA was extracted from tissue samples by Trizol regent. The integrity of total RNA was detected by agarose electrophoresis and which was quantified by NanoDrop spectrophotometer. Then, the sequencing sample library was constructed by the following steps: ribosomal RNA removal, fragmentation, first-strand complementary DNA (cDNA) synthesis, second-strand cDNA synthesis, terminal repair 3′ terminal addition, ligation, and enrichment. The libraries were sequence on an Illumina Hiseq 2500/2000 platform.

### MiRNA expression analysis

MiRNA expression levels were estimated by the TPM (transcript per million) through the following criteria: Normalized expression = mapped readcount/Total reads × 10^6^ [11]. All data were analyzed using the DESeq2 R package (1.8.3). log2FC > 1 and *p* < 0.05 were considered as the cutoff values for DEMs screening [12].

### MiRNA-mRNA network construction

Based upon results of DEM analysis and target gene prediction, the miRNA-mRNA pairs were extracted to construct the miRNA-mRNA regulatory network. Then, the regulatory network was visualized using Cytoscape_v3.5.1.

### Target gene prediction, gene ontology, and pathway enrichment analysis

Target genes of the DEMs were predicted using major online tools, including miRanda (http://miranda.org.uk/), PITA (http://genie.weizmann.ac.il/pubs/mir07/mir07_data.html), and RNAhybrid (https://bibiserv.cebitec.uni-bielefeld.de/rnahybrid/) [13]. In order to analyze the main functions of the predicted target genes for the DEMs, we performed GO analysis [14]. Moreover, KEGG [15] pathway enrichment analysis was used to find out the significant pathway of predicted target genes for the DEMs. A Go term or KEGG pathway with FDR < 0.05 was considered statistically significant. Top 10 enriched GO terms and pathways of DEMs were ranked by enrichment score (− log10(*p* value)).

## Results

### Quality assessment of sequencing data

**Fig. 1:**
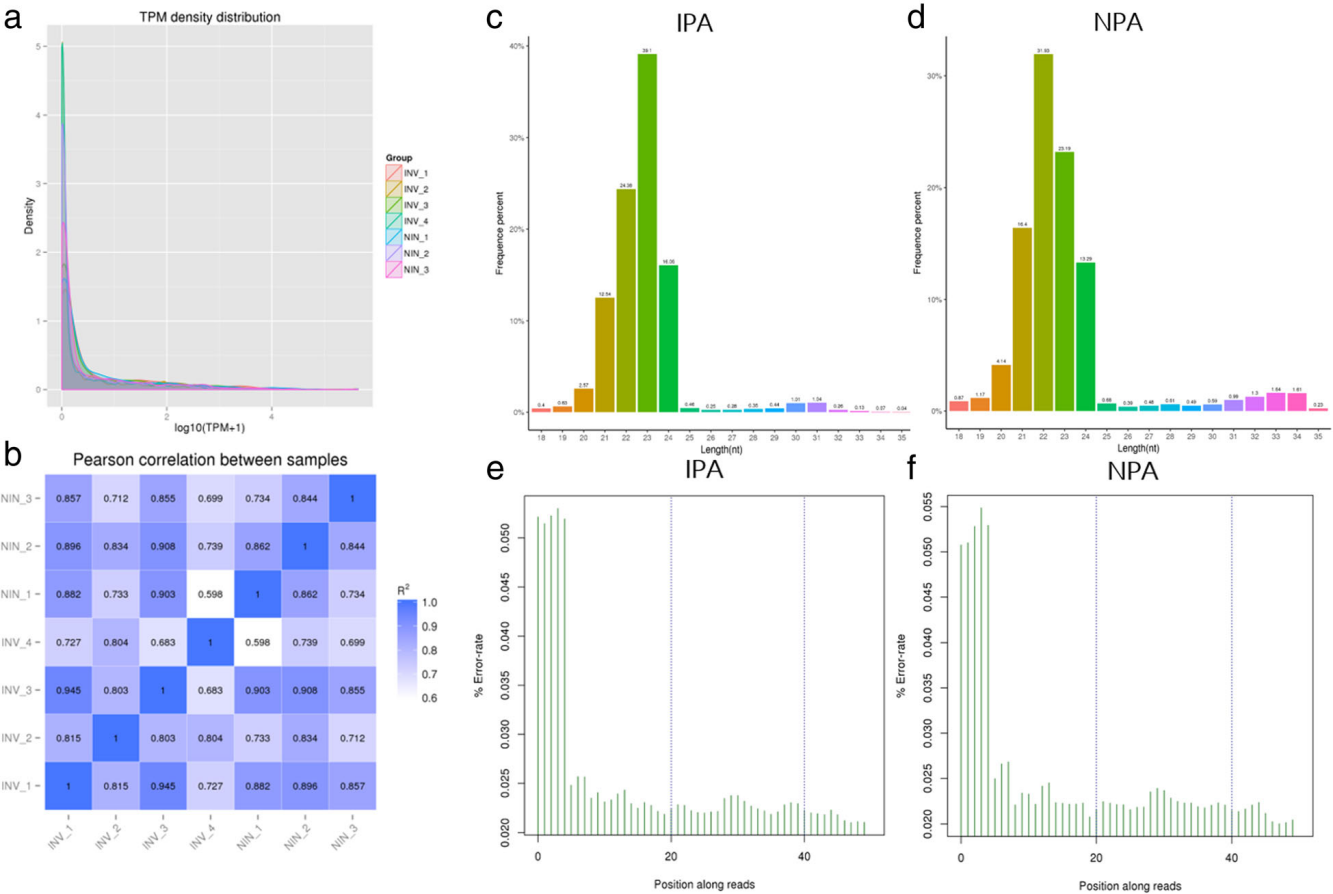
Quality control of RNA sequencing data. TPM density distribution of samples (**a**). The Pearson correlation between samples of two groups (**b**). Length distribution of total RNA fragments (**c**, **d**). Sequencing error rate distribution maps between the two groups (**e**, **f**)

The results showed the patterns of gene expression among the samples were similar, and the Pearson correlation between samples is similar in the two groups (Fig.  1a, b). In view of the fact that the length of human miRNAs is generally 19–23 nt, the analysis of the length of reads of each sample shows that 19–23 nt accounts for a higher proportion of each of 2 groups (Fig. 1c, d). And reads of the length region were included in the analysis range. In addition, by comparing and analyzing the error rate among all samples, we found that the read error rate at 19–23 nt was far less than 0.05% (Fig. 1e, f). All above results indicate the sequencing data is reliable for further bioinformation analysis.

### Apportionment and annotation of DEMs

**Fig. 2:**
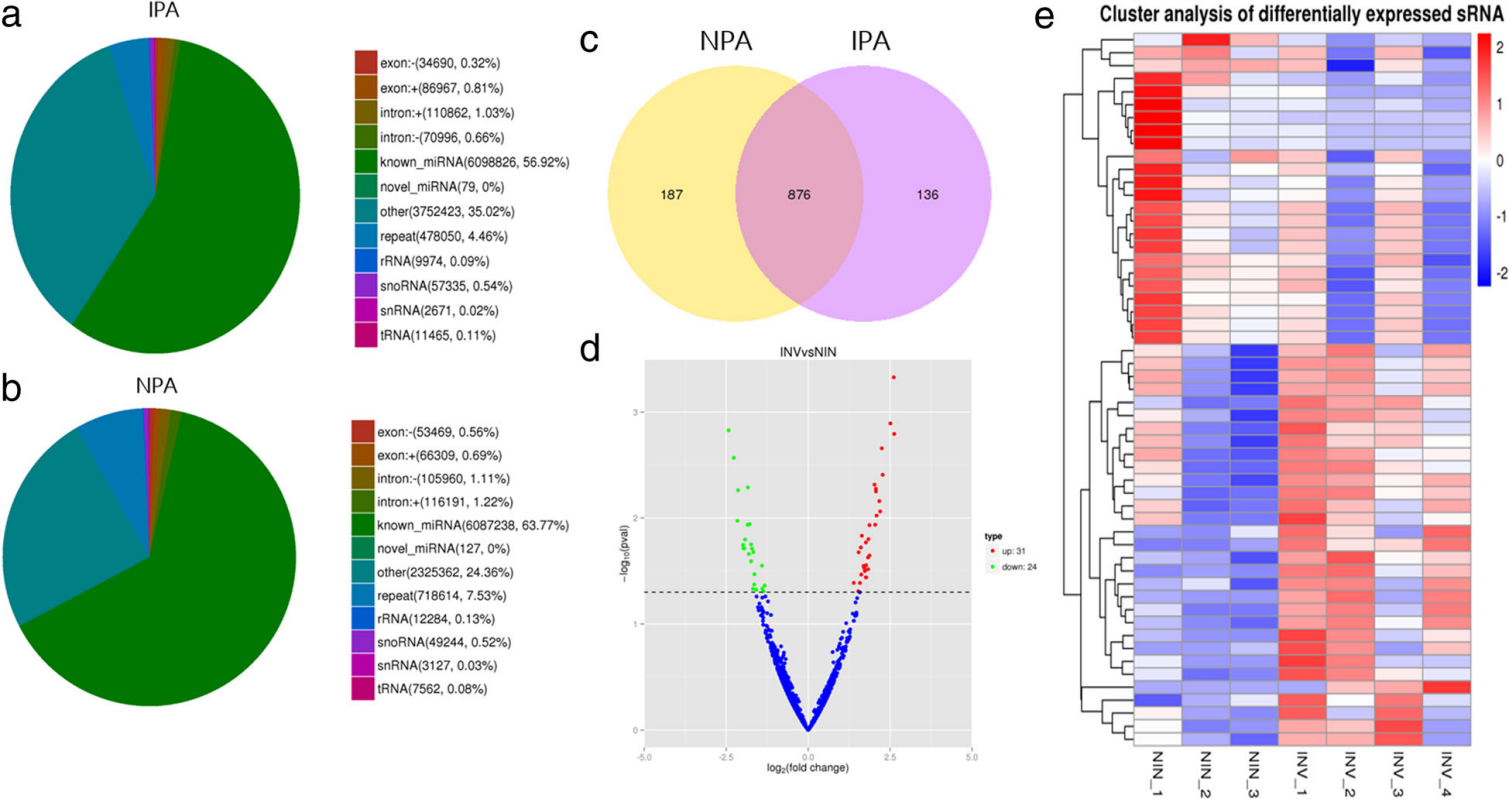
Screening of differentially expressed miRNAs. The proportional distribution of RNAs with different classification between two groups (**a**, **b**). Venn distribution of differential miRNAs, there are 136 differentially expressed miRNAs in IPA and 187 differentially expressed miRNAs in NPA (**c**). Volcanic map of differential miRNA, blue dots for no significant difference of miRNAs, red for significant upregulation of miRNAs, and green for significant downregulation of miRNAs (**d**). Cluster analysis of differentially expressed sRNAs, red for high expression miRNAs, blue for low expression miRNAs (**e**)

After summarizing and classifying the sequence reads into different RNA categories, such as miRNA, sn/snoRNA, tRNA, and rRNAs, the pie chart is drawn to annotate and classify the total reads. The proportion of known miRNAs (NIN = 63.77%, INV = 56.92%) and newly discovered miRNAs (NIN = 0.04%, INV = 0.02%) can be obtained (Fig. 2a, b).

Next, Venn diagram of the DEMs in the two groups was drawn by FunRich3.1.3. A total of 136 DEMs were found in IPA, and 187 DEMs were found in NPA (Fig. 2c).

And then, the volcano map was used to infer the overall distribution of miRNAs. ▢Log2 FC > 1 and *p* < 0.05 were used as thresholds to screen the DEMs. A total of 31 significantly upregulated and 24 significantly downregulated miRNAs were screened out (Fig. 2d).

At last, these 55 DEMs were used to construct a hierarchical clustering analysis map (Fig. 2e).

### GO and KEGG pathway analysis

**Fig. 3:**
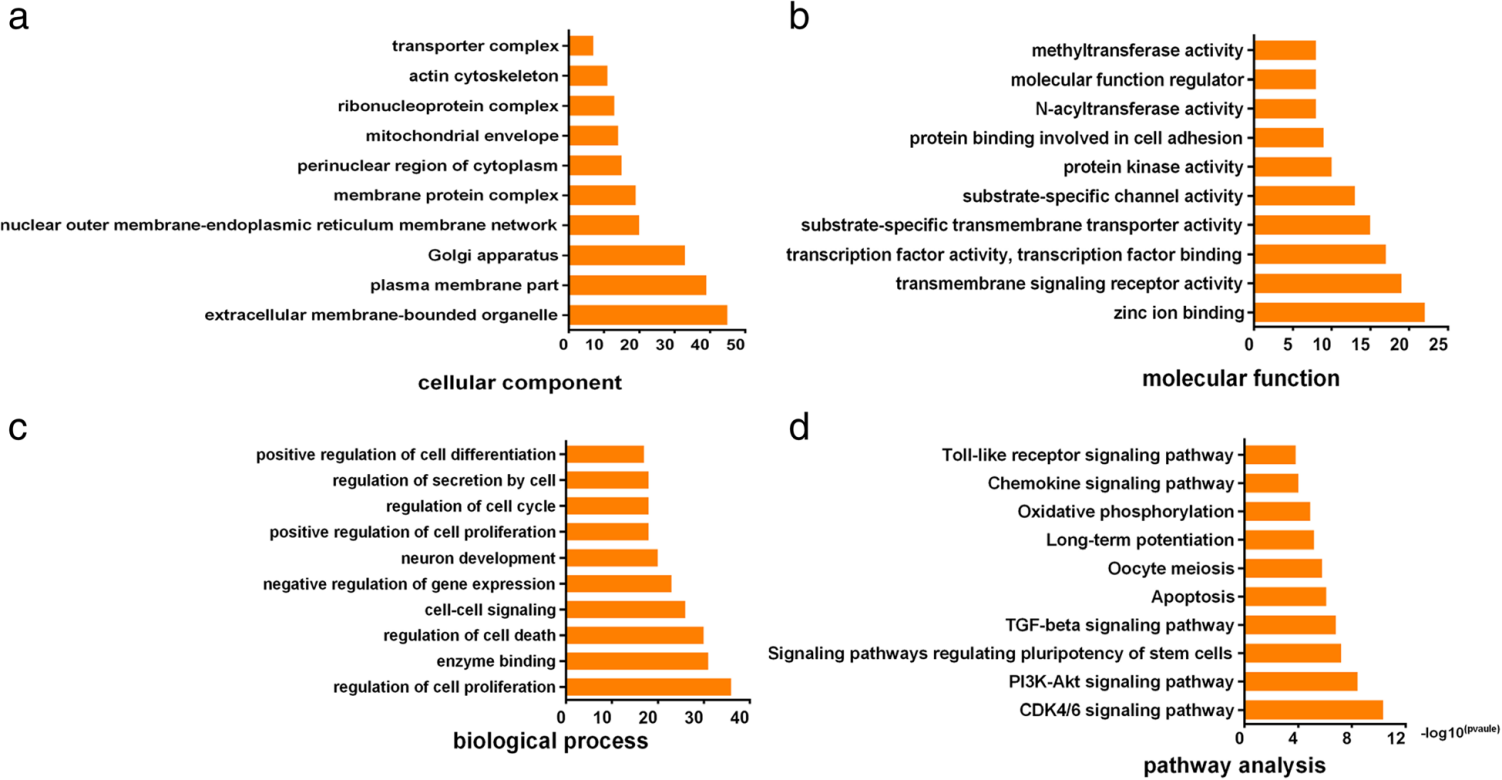
Results of GO and KEGG pathway analysis. Top 10 significant enriched BP, CC, and MF gene count by GO analysis (**a**, **b**, **c**). Top 10 significant enriched pathways by KEEG pathway analysis (**d**)

In order to explore the functions of DEMs, target genes of these miRNAs were predicted by miRanda, PITA, and RNAhybrid. GO analysis and KEGG pathway analysis were used to their target pool. As results, the results of the top 10 enriched biological process (BP) terms showed that the target gene of DEMs was associated with cell proliferation, cell cycle, and apoptosis process. The top 10 KEGG pathways showed the DEMs might be involved in CDK4/6 signaling pathway, PI3K-Akt signaling pathway, and apoptosis pathways (Fig. 3).

### MiRNA-mRNA network

**Fig. 4:**
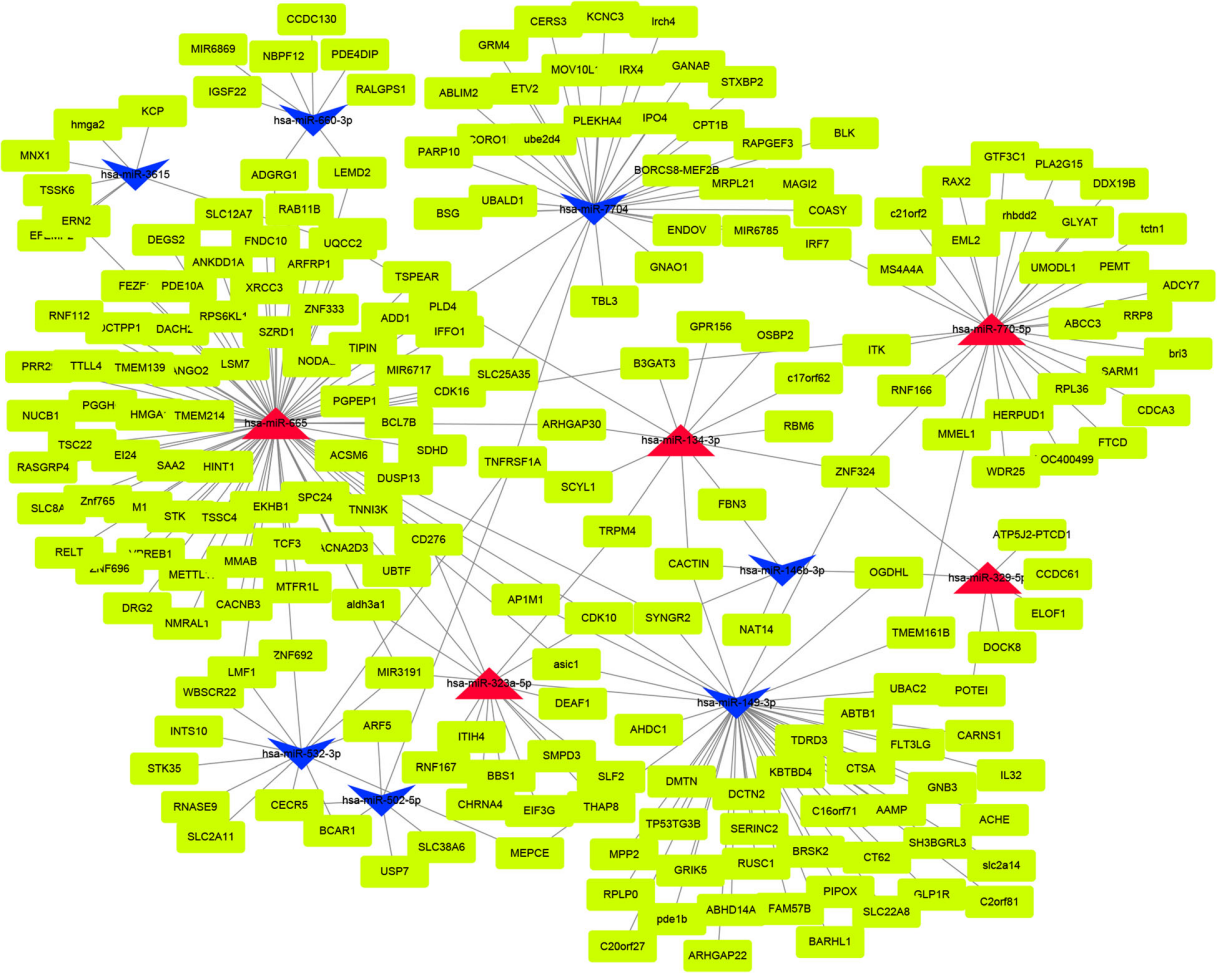
The subnetwork of differentially expressed miRNAs and targeted mRNAs, red for upregulated miRNAs, blue for downregulated miRNAs, and yellow for targeted mRNAs

Based on the DEMs and the prediction of target genes. The miRNA-mRNA network was generated by Cytoscape(v3.5.1). As we all know, hub nodes play important roles in biological networks. According to the degree of DEMs calculated by Cytoscape, a total of 12 miRNAs with higher values were identified (Table 1). Then the miRNA-mRNA subnetwork was generated, which included 5 upregulated miRNAs, 7 downregulated miRNAs, and 258 predicted target genes (Fig. 4).

**Table 1:** The node scores of miRNAs calculated by Cytoscape. The expression profiles of the upregulation and downregulation of miRNA

sRNA	Degree	INV_readcount	NIV_readcount	log2FoleChange	*p* value
has-miR-149-3p	45	0.162776612	5.71850257	− 2.1576	0.0010662
has-miR-502-5p	6	255.3260392	2064.122332	− 2.1387	0.005467
has-miR-660-3p	8	5.187907073	2664.122332	− 1.9248	0.016021
has-miR-3615	7	98.08904874	617.924121	− 1.8092	0.021926
has-miR-532-3p	13	159.302162	1183.189253	− 1.7396	0.017793
has-miR-7704	32	6.42917649	31.78715328	− 1.7119	0.019517
has-miR-146b-3p	5	96.2448944	481.269913	− 1.6392	0.033807
has-miR-665	79	4.742131447	0.001235135	1.7575	0.036171
has-miR-323a-5p	14	4554.136246	870.3564614	1.7582	0.017047
has-miR-134-3p	12	20.80177896	2.293937139	1.8693	0.022669
has-miR-329-5p	7	963.6719203	135.5546582	2.065	0.005295
has-miR-770-5p	30	18.03698596	0.409414746	2.625	0.001605

## Discussion

Although PAs are classified into multiple subgroups based on the histological structure, pathological types, and hormone secretion [16, 17], the definition of clinically IPAs currently differs in the literature. In according to the 4th edition of the WHO classification of endocrine tumors published in 2017, the invasiveness of PAs can be evaluated through the tumor proliferative capacity by mitotic count and Ki-67 index [18], indicating that the tumor proliferation capacity is closely related to invasion of PAs.

MiRNAs, mediate post-transcriptional regulation, play an important role in epigenetic regulation. Its precursor is cleavage by Dicer enzyme, and then combined with AGO protein and other components to form RISC (RNA immuno-silencing complex) to play a key role in silencing or degradation of target mRNA [19, 20]. Some researchers have reported the regulating functions of miRNAs in PAs [21]. Upregulated miR-34a can significantly inhibit the proliferation of PA cells GH4C1 and promote apoptosis by regulating SOX7 [22]. Overexpression of miR-16 inhibits the proliferation of PA cells HP75 and promotes apoptosis by targeting HMGA2 expression [23]. However, the comprehensive and specific effects of miRNAs in PA invasion behavior are still rarely reported [24]. Thus, we recognized 55DEMs in IPAs by RNA sequencing analysis. Then, according to Cytoscape calculation, a miRNA-mRNA co-expression network including 5 upregulated miRNAs, 7 downregulated miRNAs, and 258 predicted target genes was generated in IPAs.

To further understand the potential function of miRNAs, GO and KEEG pathway analysis were applied for analyzing their possible biological functions and the signaling pathways in IPAs. The outcome of GO analysis showed that the target genes for the DEMs in IPAs were enriched for genes associated with cell proliferation, cell cycle, and apoptosis process, which is consistent with the active proliferation characteristics of IPA cells. Meanwhile, KEEG pathway analysis showed that the DEMs might be involved in CDK4/6 signaling pathway, PI3K-Akt signaling pathway, and apoptosis pathways. It is noted that the enriched terms of target genes for the DEMs from KEEG analysis were consistent with GO analysis results, and they are all closely related to cell proliferation. As mentioned above, tumor proliferation capacity is the most important feature of IPAs.

Additionally, the miRNA-mRNA network was constructed and the outcome showed that some hub miRNAs play important roles in IPAs. The hub miRNAs were identified by the degree of DEMs calculated by Cytoscape. Intriguingly, we found that these miRNAs have similar biological functions. For instance, hsa-miR-665, the most upregulated node in IPAs, can promote tumor cell proliferation or cell cycle progression in hepatocellular carcinoma [25]. In contrast, it was found that overexpression of has-miR-149-3p, the most downregulated node in IPAs, can inhibit the proliferation and invasion of tumor cells in bladder cancer and renal epithelial cell carcinoma [26].

## Conclusions

In this study, we identified the DEMs related to the invasive behavior of PAs. Further study is needed to confirm the exact relationship between DEMs and invasive behavior of PAs and clarify the molecular mechanism of miRNA affecting the invasion of PAs.

## Data Availability

Please contact author for data requests.

## Abbreviations

BP: Biological process
DEM: Differential expression miRNA
GO: Gene ontology
IPA: Invasive pituitary adenoma
KEEG: Encyclopedia of Genes and Genomes
MiRNA: MicroRNA
NPA: Non-invasive pituitary adenoma
PA: Pituitary adenoma
